# Case report: Ruxolitinib as first-line therapy for secondary hemophagocytic lymphohistiocytosis in patients with AIDS

**DOI:** 10.3389/fimmu.2022.1012643

**Published:** 2022-10-03

**Authors:** Xiang Liu, Xueling Zhu, Xiaotang Zhou, Yirui Xie, Dairong Xiang, Zhikai Wan, Ying Huang, Biao Zhu

**Affiliations:** The Department of Infectious Diseases, State Key Laboratory for Diagnosis and Treatment of Infectious Diseases, National Clinical Research Center for Infectious Diseases, Collaborative Innovation Center for Diagnosis and Treatment of Infectious Diseases, The First Affiliated Hospital, School of Medicine, Zhejiang University, Hangzhou, China

**Keywords:** ruxolitinib, hemophagocytic lymphohistiocytosis, acquired immune deficiency syndrome, cytokines, inflammation, therapeutics

## Abstract

**Background:**

Hemophagocytic lymphohistiocytosis (HLH) is a fatal immunological syndrome resulting from excessive production of inflammatory cytokines. The conventional therapies for HLH, which are based on cytotoxic agents, are not always efficacious and safe, especially in patients with severe immunodeficiency. Ruxolitinib, a strong inhibitor of Janus kinase (JAK) 1/2, has already been evaluated as salvage and first-line therapy for HLH. Despite its promising efficacy and tolerability in the treatment of secondary HLH, the efficacy and safety of ruxolitinib in HLH patients with HIV infection remain to be investigated.

**Case presentation:**

Two men (ages: 45 and 58 years) both presented at our hospital with a high fever. They were found to be HIV-positive with severe immunodeficiency and opportunistic infections. Their laboratory tests showed severe pancytopenia, hypofibrinogenemia, hypertriglyceridemia, and increased levels of inflammatory factors and ferritin. Hemophagocytosis was found in the bone marrow, and abdominal computed tomography or ultrasonography showed splenomegaly. Both patients were diagnosed with infection-induced HLH due to severe immunodeficiency. Given they were both highly immunocompromised, we chose ruxolitinib as a first-line treatment alternative to cytotoxic chemotherapy. Rapid remission of clinical symptoms and normalization of laboratory parameters were achieved after ruxolitinib therapy. Neither patient had any associated adverse drug reactions or other laboratory abnormalities. Both patients were eventually discharged and ruxolitinib was discontinued as their disease alleviated, and they did not show signs of relapse during the 3- and 5-month of follow-up examinations.

**Conclusion:**

We described two cases of AIDS-related secondary HLH treated with ruxolitinib. Our cases highlight the feasibility of using ruxolitinib as a first-line therapy in patients with HIV infection and secondary HLH. Nevertheless, the safety and efficacy of this novel treatment need to be evaluated in large clinical trials in the future.

## Introduction

Hemophagocytic lymphohistiocytosis (HLH) is a rare hyperinflammatory syndrome driven by overactive T cells and macrophages that secrete large amounts of inflammatory cytokines, including interferon (IFN)-γ, interleukin (IL)-1-beta, IL-2, IL-6, IL-10, IL-18, and tumor necrosis factor (TNF) (1, 2). The pathophysiology and clinical symptoms of HLH are caused by the release of cytokines, which, if ignored, can result in multi-organ failure and death. Adult HLH is a highly fatal illness with a death rate higher than 40% and is more commonly observed as secondary to underlying infectious, neoplastic, autoimmune, and immunodeficiency etiologies (3, 4) Current therapeutic regimens for adult HLH derived from the HLH-2004 protocol, which are based on etoposide, dexamethasone, and cyclosporin A (CSA) (5). However, concerns have been raised about the effectiveness and safety of these cytotoxic and immunosuppressive treatments, which may compromise the clearance of pathogenic microorganisms, especially in immunodeficient patients (2, 6, 7). AIDS-related HLH is mostly induced by severe immunodeficiency and co-infection. Therefore, more caution should be exercised while administering cytotoxic and immunosuppressive agents to affected patients. Effective alternatives would be tailored therapeutic strategies that target multiple cytokines to alleviate hyperinflammation.

The JAK-STAT-dependent signaling pathway promotes excessive production of cytokines by activated T cells and macrophages (8). Based on the crucial functions in transferring the signals induced by cytokines, patients with HLH might benefit from JAK inhibition as a therapeutic approach. Ruxolitinib is a powerful and highly bioavailable oral JAK1/2 inhibitor that has been licensed for treating autoimmune disorders and myeloproliferative neoplasms (9, 10). It has been shown to be efficacious for treating HLH in murine models and in several clinical studies (8, 11, 12). However, not enough studies have confirmed the feasibility of using ruxolitinib in treating AIDS-related HLH. Here, we report our experience of treating two cases of AIDS-related HLH by using ruxolitinib as a first-line treatment.

## Case presentation

### Case one

**Figure 1:**
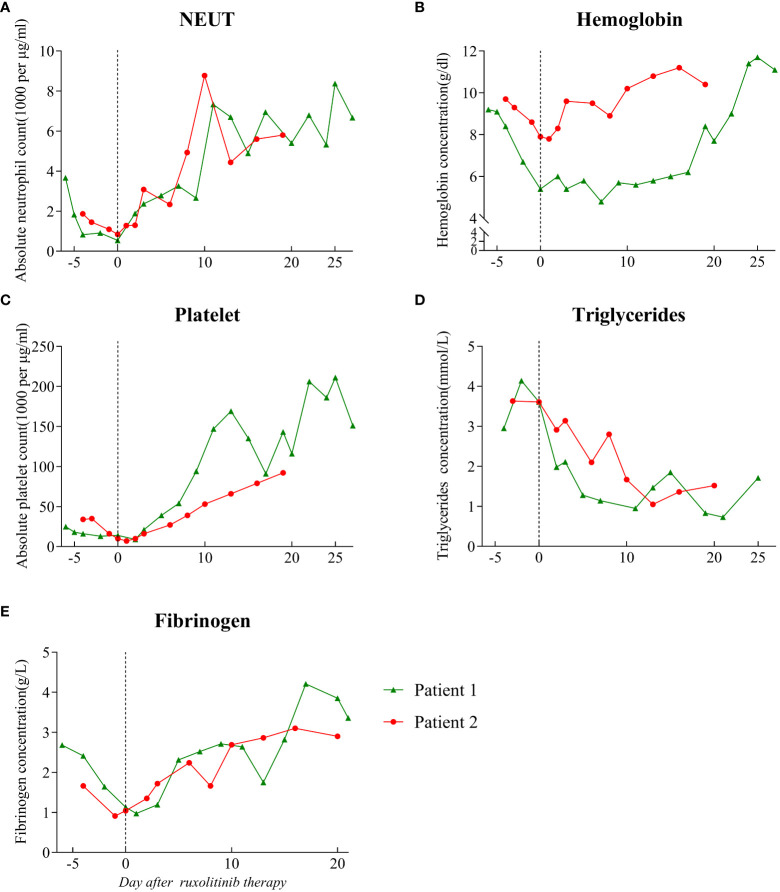
Hematological parameters prior to and following the start of ruxolitinib treatment. The dotted line on the graph indicates the day on which ruxolitinib treatment was started. **(A)** Absolute neutrophil count. **(B)** Hemoglobin level. **(C)** Absolute platelet count. **(D)** Triglyceride level. **(E)** Fibrinogen level. NEUT: neutrophil.

**Figure 2:**
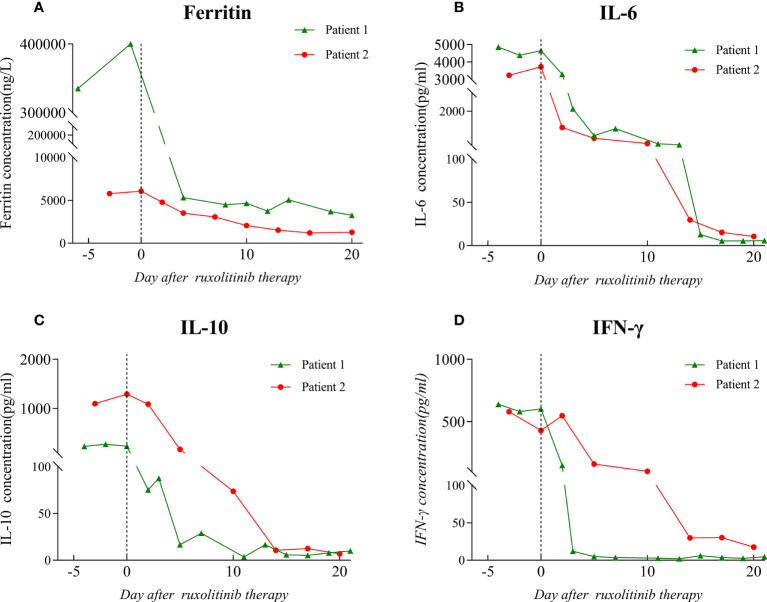
Ferritin and cytokine level before and after the initiation of ruxolitinib treatment. The dotted line on the graph indicates the day on which ruxolitinib treatment was started. **(A)** Ferritin level. **(B)** IL-6 level. **(C)** IL-10 level. **(D)** IFN-γ level. IL: interleukin; IFN: interferon.

A local hospital referred to our hospital a 45-year-old man with recurrent high fever and cough for 2 weeks. His symptoms were as follows: weakness, fever, cough, appetite loss, and weight loss of nearly 10 kg in the previous few weeks. The patient is a male homosexual and denied any other past medical, surgical history and family history including HLH. Lung computed tomography (CT) performed at the local hospital revealed dispersed inflammation in both lungs, left upper lung occupancy, and obstructive inflammation. Laboratory assessment showed HIV antibodies, pancytopenia, and elevated levels of liver function markers. After admission, the patient developed a daily high-spiking fever of 38.5°C to 40°C with a diffuse red rash and was extremely debilitated. Initial laboratory results showed aggravation of pancytopenia (neutrophil count, 1.83 to 0.54 × 10^9^/L; hemoglobin level, 84 to 54 g/L, platelets 16 to 9 × 10^9^/L), hypofibrinogenemia (0.97 g/L), hypertriglyceridemia (4.14 mmol/L), D-dimer level of >88000 µg/L, and ferritin level of 400 µg/L(**Figures 1A–E**, **2A** and **Table 1**). He also had severe hypoalbuminemia (18.9 g/L), normal stable renal function, mild jaundice, and transaminitis (**Table 1**). His CD4+ cell count was 6/µl and Epstein-Barr virus(EBV) and human cytomegalovirus (HCMV)-DNA loads were 35300 and 3400 copies/ml, respectively. Inflammatory marker levels were elevated, with procalcitonin and C-reactive protein (CRP) levels of 11.4 ng/mL and 145.47 mg/L, respectively. Moreover, the plasma inflammatory cytokine assay revealed IL-6(normal range, 0.1-2.9 pg/ml), IL-10(normal range, 0.1-5.0 pg/ml), and IFN-γ (normal range, 0.1-18pg/ml)levels of 4853.22, 229.81, and 639.54 pg/ml, respectively (**Figures 2B–D**). Broad-spectrum antibiotic treatment was initiated to control infection. Other therapies included prophylactic cotrimoxazole for PCP prophylaxis and antiviral therapy was considered to manage the HCMV infection. Three days after admission, bone marrow aspiration was performed and hemophagocytosis was found in the bone marrow. Ultrasonography showed splenomegaly (thickness of 4.5cm). On hospital day 4, blood cultures were found to be positive for *Talaromyces marneffei*. The patient developed distributive shock and convulsive seizures. Taken together, the patient fulfilled at least six criteria of the HLH 2004 protocol (13) and was diagnosed with HLH and septic shock due to disseminated *Talaromyces marneffei* infection.

**Table 1:** Laboratory parameters before and after ruxolitinib treatment.

	Before ruxolitinib therapy	At discharge
**Case one**		
CRP (normal range,0-8mg/ml)	145.47	5.40
D-dimer (0-700 μg/L)	>88000	450
Procalcitonin (0-0.5 ng/ml)	11.40	0.25
ALT (9-50 U/L)	194	18
AST (14-40 U/L)	740	13
plasma albumin(40-55g/L)	18.9	37.9
EPI-cr	80.3	105.2
**Case two**		
C-reactive protein	137.60	14.5
D-dimer	32960	735
procalcitonin	1.21	0.04
ALT	68	22
AST	116	38
plasma albumin	23.2	35.2
EPI-cr	50.1	104.6

CRP, C-reactive protein; EPI-cr, Epidemiology Collaboration- creatinine.

The patient was started on antifungal, immunoglobulin, and glucocorticoid therapies as well as supportive treatment. Given his critical clinical condition and severe immunodeficiency profile, chemo-immunotherapy was considered ineffective and poorly tolerated (6, 14). On day 6 of hospitalization, ruxolitinib 15 mg twice daily was considered an option upon extensive discussion with a hematologist and the patient. After starting ruxolitinib administration, blood was analyzed for hematological and biochemical parameters every other day, and no side effects were noted. On the fifth day after ruxolitinib therapy, his platelet and neutrophil counts increased to 39 × 10^9^/L and 2.78 × 10^9^/L, respectively, and ferritin, CRP, IL-6, IL-10, and IFN-γ levels decreased to 5326.7 ng/ml, 5.4 mg/L, 636.35 pg/ml, 16.57 pg/ml, and 4.87 pg/ml, respectively (**Figures 1**, **2**). After 11 days of therapy, total bilirubin, alanine aminotransferase (ALT), aspartate aminotransferase (AST), triglyceride, and plasma albumin levels of 14.9 µmol/L, 68 µmol/L, 40 µmol/L, 0.95 mmol/L, and 32.8 g/L, respectively, were noted. A second chest enlargement CT revealed a decreased left upper lung lesion; furthermore, there was not enough evidence for it to be considered cancerous. The patient’s clinical and laboratory indicators largely normalized after 21 days of ruxolitinib therapy (**Figures 1**, **2**). Ultrasound showed that the spleen thickness dropped to 3.7 cm. Combination antiretroviral therapy (cART) was started before the patient was discharged (**Table 1**). Further, the patient was very gradually switched from amphotericin B to oral itraconazole, and the dose of ruxolitinib was gradually decreased and eventually discontinued. Disease recurrence was not noted at the 5-month follow-up examination.

### Case two

A 58-year-old man who had been experiencing chills, fatigue, and recurring fever for 10 days visited our hospital. He had been diagnosed to have AIDS, non-tuberculous mycobacteria (NTM) infection, and infection with *Talaromyces marneffei* 3 months ago. Since then, he had received highly active antiretroviral therapy, anti-NTM therapy, and antifungal medication. Furthermore, he had a 10-year history of diabetes and had no personal or family history of HLH. The patient claimed to have lost 10 kg in the previous 6 months. After being admitted, the patient experienced frequent episodes of high fever, with a maximum temperature of 39.8°C, ongoing chest pressure, and low mental well-being. He was found to have neutropenia (neutrophil count: 0.84 × 10^9^/L), anemia (hemoglobin level: 78 g/L), and severe thrombocytopenia (platelet count: 7 × 10^9^/L) (**Figure 1A–C**). The levels of IL-6, IL-10, and IFN-γ were markedly high (**Figure 2B–D**). His triglyceride, fibrinogen, ferritin and D-dimer levels were 3.63 mmol/L, 0.91 g/L,6070 ng/mL, and 32960 µg/L, respectively (**Figures 1D, E** and **Figure 2A**). Moreover, he had severe hypoalbuminemia, a CD4+ lymphocyte count of just 8/µl, and renal failure (**Table 1**). Hepatosplenomegaly was detected on an abdominal CT. Importantly, bone marrow aspiration testing revealed hemophagocytosis. Based on HLH-2004 diagnostic criteria, the patient’s symptoms were compatible with those of HLH. We were unable to determine the cause of the infection at first because the blood bacterial culture was negative. However, the next-generation sequencing (NGS) revealed the herpes virus 4 (EBV)as a culprit. EBV-DNA was 835000 copies/ml. We believe that the patient had an outbreak of HLH due to EBV activation on the basis of poor immune reconstitution.

In light of the patient’s extremely poor immune status and renal failure, ruxolitinib 15 mg PO bid was started, and empiric antibiotics were started to manage any potential pathogen infections along with continuous administration of cART, anti-NTM therapy, and antifungal medication. After 3 days of ruxolitinib treatment, he no longer had a fever, and his strength and appetite had started to return. His neutrophil and platelet counts improved to 2.34 × 10^9^/L and 27 × 10^9^/L (**Figure 1A, C**), respectively, and CRP level decreased to 34.3 mg/ml. His ferritin level dropped to 3062 ng/mL, fibrinogen level was 2.24 g/L, and D-dimer level was 7350 µg/L after 6 days of treatment. After therapy for 14 days, his hemoglobin level and platelet count increased to 108 g/l and 66 × 10^9^/L, respectively, and neutrophil count, kidney function, and triglyceride level normalized (**Figure 1**). The levels of HLH-related cytokines IL-6, IL-10, and IFN-γ decreased to 29.85, 10.7, and 29.85 pg/ml, respectively (**Figure 2**). Furthermore, ultrasound showed that hepatosplenomegaly resolved (thickness of 3.5cm). After the cART regimen was modified and the patient’s indicators markedly improved, the patient was discharged in a stable condition 21 days after being admitted (**Table 1**). Antifungal and anti-NTM medications were continued while ruxolitinib was gradual tapered and discontinued after 4 weeks. No signs of recurrence were observed at 3 months after discharge from the hospital.

## Discussion and literature review

Here, we report cases of two patients with HLH secondary to AIDS who were successfully treated with ruxolitinib as the first-line therapy. After ruxolitinib treatment, the clinical symptoms of both patients improved rapidly, including resolution of cytopenia and splenomegaly, recovery of liver and renal function, significant improvements in fibrinogen and ferritin levels, and decrease in the levels of HLH-associated cytokines (IL-6, IL-10, and IFN-γ). Ruxolitinib was well tolerated and there were no severe adverse effects in either patient. The patients were eventually discharged and ruxolitinib was discontinued, which preliminarily confirmed that ruxolitinib is feasible as a first-line treatment for HLH secondary to AIDS and is safe and well tolerated in patients with severe immunodeficiency.

Etoposide and corticosteroids are the underlying drugs for the conventional therapeutic regimens for secondary HLH, and etoposide was shown to selectively eliminate activated T cells in murine models (15, 16). Both of our patients presented with severe immunodeficiency and extremely low CD4^+^ counts. Therefore, we exercised extreme caution when using etoposide and other immunosuppressive drugs. Pulmonary malignancy was initially suspected in the first patient; however, he was eventually confirmed to be infected with *Talaromyces marneffei*. Antifungal and glucocorticoid therapies were initiated as soon as the diagnosis was established. The patient’s fever could be controlled with the treatment; however, severe cytopenias, especially thrombocytopenia, persisted. Despite the potential risk of thrombocytopenia exacerbation with ruxolitinib (8, 17), the patient’s platelet counts greatly increased and other evaluated parameters improved significantly after ruxolitinib treatment. The second patient had been on cART for almost 3 months at the time of onset of HLH, making it easy for his condition to be associated with immune reconstitution inflammatory syndrome (IRIS). However, his CD4^+^ count was only 8/µl, and therefore, we rejected the diagnosis of IRIS. Because of our experience with the first case, we initiated ruxolitinib treatment as soon as possible. The patient became afebrile and showed a significant improvement in inflammatory marker levels and organ function. It should be noted that ruxolitinib alone and not corticosteroid treatment was administered for HLH management, which helped avoid the side effects of corticosteroids.

Ruxolitinib inhibits activation of JAK/STAT signaling pathways, where most HLH-related cytokines (IFN-γ, IL-2, IL-6 and IL-10) converge on (11). By blocking this route, cytokine signaling and inflammation are reduced. It was demonstrated in preclinical models (11, 18), and several clinical studies (8, 12, 19, 20). Our cases found that ruxolitinib treatment greatly decreased the levels of CRP, PCT and cytokines related to HLH. The pancytopenia and other laboratory parameters improved by decreasing cytokine production and inflammation. This is a possible explanation for the rapid control of the patient’s symptoms after ruxolitinib therapy. Additionally, ruxolitinib was found to improve the clinical condition within 24–48 hours of its administration, and the majority of laboratory values normalized within 7–30 days. All were similar to the previous findings. The splenomegaly resolved after use of ruxolitinib in our cases report, while ruxolitinib’s effect in shrinking the enlarged spleen is not satisfactory in a previous publication (21). The two patients were treated with ruxolitinib for less than 2 months, which is shorter than the treatment time reported previously (22, 23). Not enough studies have been conducted to determine the optimal duration of ruxolitinib treatment. But some studies demonstrated ruxolitinib was allowed to be tapered off gradually and then terminated (24), which was also true in our study subjects. No disease recurrence was noted in either case at the 3-month follow-up. Needless to say, continued observation is necessary.

The treatment was well tolerated in our cases, and no serious side effects occurred during treatment and follow-up, indicating that ruxolitinib is safe for the management of AIDS-related HLH. A recent randomized controlled trial conducted by Marconi et al. also demonstrated that ruxolitinib was safe and well tolerated in HIV-positive patients with virologic suppression and markedly decreased the levels of immune activation markers (25). Interestingly, some studies found that ruxolitinib inhibited HIV-1 replication and virus reactivation *in vitro*, and targeted the gamma-C receptor cytokine to affect the magnitude of the HIV reservoir in all memory CD4 T cell subsets *in vitro* and ex vivo (26, 27). However, there were no significant changes in the HIV reservoir after 5 weeks of ruxolitinib therapy in the study conducted by Marconi et al. (25). Ruxolitinib treatment for 5 weeks may not be sufficient to reduce the HIV reservoir, and a longer treatment duration is needed to achieve changes in the HIV reservoir. Overall, ruxolitinib was safe and well tolerated in patient with HIV-related HLH. Further investigation of ruxolitinib in populations with HIV-1 infection is worthwhile.

Previously, ruxolitinib was thought to be effective and well tolerated as salvage therapy when administered twice daily at doses of 2.5–25 mg in cases of relapsed/refractory forms of HLH. Some researchers showed the potential of ruxolitinib as a first-line treatment for secondary HLH. Ruxolitinib was used as first-line treatment for two adult patients with HLH caused by rheumatoid arthritis and *Histoplasma capsulatum* infection (6, 14). Ahmed et al. (8) presented the outcomes of an open-label, prospective, single-center study in which ruxolitinib was effective as a first-line treatment for five persons with HLH. In a recent report of investigation conducted in a pediatric HLH cohort, 12 patients with newly diagnosed secondary HLH were treated with ruxolitinib as the first-line treatment, and 10 patients (83%) showed improvement at 28 days with no significant adverse effects (28). However, the use of ruxolitinib in patients with AIDS remains rare. Gálvez Acosta et al. (29) reported a case of a Cameroonian male patient with HIV-associated hemophagocytic syndrome in which first-line treatment with ruxolitinib was successful. This is the only reported case of successful first-line treatment with ruxolitinib in an HIV-1–infected patient to date.

Although we reported two cases of ruxolitinib as a first-line treatment option for secondary HLH, there are still some challenges for the use of ruxolitinib as a first-line treatment for AIDS-related HLH. Firstly, thrombocytopenia was the major side effect of ruxolitinib for hematologic disorders (17). And whether ruxolitinib further aggravated thrombocytopenia was the biggest concern during our treatment. Secondly, ruxolitinib is not widely recommended as a first-line therapy for secondary HLH. It would be considered to delay patient treatment if did not achieve the desired outcome. Therefore, more studies are required to validate the safety and effectiveness of ruxolitinib as a first-line treatment for HLH in HIV-positive patients.

## Conclusion

Our successful treatment of two patients with AIDS-associated HLH by using ruxolitinib instead of chemo-immunotherapy provides valuable insight for the use of ruxolitinib as a first-line treatment option for AIDS-associated HLH. It also would be beneficial to the broader community to further emphasize that the cytopenias in HLH are frequently caused by the cytokine storm and resolve with cytokine-based therapy.

## Data availability statement

The original contributions presented in the study are included in the article/Supplementary Material. Further inquiries can be directed to the corresponding author.

## Ethics statement

Written informed consent was obtained from two patients for the publication of this case report.

## Author contributions

XL and XTZ provided clinical specimens and information. XL and XLZ performed the data analysis and drafted the manuscript. BZ and YRX designed the study. DRX, ZKW and YH participated in the study design and coordinated the drafting of the manuscript. All the authors read and approved the final manuscript. 

## Funding

This work was supported by the National Key R&D Program of China [grant number 2021YFC2301900- 2021YFC2301901]; and the National Special Research Program for Important Infectious Diseases [grant number 2017ZX10202102].

## Conflict of interest

The authors declare that the research was conducted in the absence of any commercial or financial relationships that could be construed as a potential conflict of interest.

## Publisher’s note

All claims expressed in this article are solely those of the authors and do not necessarily represent those of their affiliated organizations, or those of the publisher, the editors and the reviewers. Any product that may be evaluated in this article, or claim that may be made by its manufacturer, is not guaranteed or endorsed by the publisher.
